# The role of impaired brain perfusion in septic encephalopathy

**DOI:** 10.1186/s13054-018-2299-z

**Published:** 2019-02-19

**Authors:** Lucia Rivera-Lara

**Affiliations:** 1 Department of Neurology, The Johns Hopkins University School of Medicine, 600 N Wolfe Street, Phipps 455, Baltimore, MD 21287 USA; 2 Department of Anesthesiology and Critical Care Medicine, The Johns Hopkins University School of Medicine, 600 N Wolfe Street, Phipps 455, Baltimore, MD 21287 USA

Sepsis-associated encephalopathy (SAE), also known as sepsis-associated brain dysfunction (SABD) or sepsis-associated delirium, is an acute condition that has been reported to affect up to 76% of patients with sepsis [1]. Furthermore, patients with SAE have higher in-hospital mortality than do patients who have sepsis without SAE (62.4% versus 23.4%, *P* <0.0001) [2, 3].

For decades, it has been thought that the brain dysfunction in SAE is a consequence of the inflammatory response to a systemic infection; however, evidence indicates that sepsis survivors have long-term cognitive impairment, lower quality of life, and structural brain lesions that include hippocampal atrophy, white matter disease, and ischemic strokes [4–6]. These long-term sequelae suggest that SAE may be secondary to microcirculatory dysfunction in the brain with insufficient blood supply. A study in animals showed that somatosensory amplitudes and related evoked flow velocity responses decreased in rats with severe sepsis when cerebral hyperemia was just beginning to develop. The activation flow coupling was impaired in severe sepsis, and the decrease in evoked flow velocity preceded that of somatosensory amplitudes by nearly 2 h, suggesting inappropriate blood supply of the activated brain area [7]. Furthermore, in a prospective study of 112 patients with sepsis, Yao et al. [3] compared brain injury biomarker levels in patients who did and those who did not develop SAE. The authors found that both S100b and neuron-specific enolase were significantly higher in patients with SAE than in those without SAE. These results suggest possible neuronal and glial injury during SAE that could be secondary to hypoperfusion.

Impaired cerebral autoregulation (CA) has also been reported in up to 60% of patients with sepsis on day 1; interestingly, impaired CA at day 1 is associated with SAE at day 4 [1]. A recent study published in *Critical Care* with a larger sample size re-emphasizes this issue. Crippa and co-authors completed a prospective study of 100 patients with sepsis in two European intensive care units (ICUs). The aim of this study was to evaluate the association between altered CA and the occurrence of SABD. CA was measured by using transcranial Doppler. Mxa was calculated with MATLAB (MathWorks, Natick, MA, USA) as the Pearson’s correlation coefficient between the averaged mean arterial pressure (MAP) and flow velocity. Patients were monitored for only 10 min, 48 h after diagnosis of sepsis. In this study, 50% of patients had impaired CA. SABD was diagnosed in 57% of patients, and those with SABD had longer ICU length of stay and greater ICU mortality than patients without SABD. SABD was more common in patients with altered CA than in those with intact CA.

Prior studies of CA in sepsis have included rather small numbers of patients (<40); however, they have shown a consistent pattern in which patients with SAE have more impaired CA than those patients without it [1, 8, 9]. Impairment of CA in sepsis likely has two causes. The first occurs when MAP falls below the patient’s lower limit of CA. Modern data on autoregulation in adults have shown that the lower limits of autoregulation may vary from 40 to 90 mm Hg [10]. A recent study investigating higher blood pressure targets for patients with septic shock showed that the subset of patients with chronic hypertension randomly assigned to the higher MAP goal of 80 to 85 mm Hg required less renal replacement therapy than similar patients who were randomly assigned to the MAP goal of 65 to 70 mm Hg [11]. The latter study suggests that when MAP has dropped below the lower limit of CA, renal perfusion is also at risk. This link between kidney and brain autoregulation has been proposed before [12].

The second reason for impaired CA in sepsis, and actually the major confounder when monitoring cerebral hemodynamics in patients with sepsis, is the vascular dysfunction triggered by the systemic inflammatory process. Disruption of the blood–brain barrier, nitric oxide accumulation, and microvascular damage are only some potential causes [13–15]. Taccone et al. [13] found that, in septic animals, cerebral functional capillary density and the proportion of small perfused vessels decreased significantly at the onset of septic shock when compared with non-septic animals. Concomitantly, brain oxygen tension (PbO_2_) decreased and lactate/pyruvate ratio increased. At 18 h, when shock was present, animals with MAP of less than 65 mm Hg (n = 6) had similar functional capillary density, proportion of small perfused vessels, and PbO_2_ values but a significantly higher lactate/pyruvate ratio when compared with animals whose MAP was 65–70 mm Hg (n = 4) [13]. Therefore, both of these alterations (systemic inflammatory process and hypotension) likely play an important role in the pathogenesis of brain dysfunction during sepsis.

To move forward and find the true answer to the question of whether SAE is a consequence of brain hypoperfusion secondary to impaired CA, future studies need to standardize definitions of brain dysfunction, delirium, and encephalopathy in the ICU. Second, a more comprehensive set of neuromonitoring tools should be applied to study the cerebral hemodynamics in these patients. Lastly, we need to study the brain more comprehensively, as SAE is a diagnosis of exclusion. Therefore, we need brain imaging and complete neurological assessments to rule out other brain disorders.

## Abbreviations

CA: Cerebral autoregulation
ICU: Intensive care unit
MAP: Mean arterial pressure
PbO_2_: Brain oxygen tension
SABD: Sepsis-associated brain dysfunction
SAE: Sepsis-associated encephalopathy
